# Co-designing strategies to improve advance care planning among people from culturally and linguistically diverse backgrounds with cancer: iCanCarePlan study protocol

**DOI:** 10.1186/s12904-024-01453-z

**Published:** 2024-05-18

**Authors:** Ashfaq Chauhan, Upma Chitkara, Ramya Walsan, Ursula M. Sansom-Daly, Elizabeth Manias, Davinia Seah, Angie Dalli, Nadine El-Kabbout, Thit Tieu, Mashreka Sarwar, Misbah Faiz, Nancy Huang, Vitor Moraes Rocha, Abhijit Pal, Reema Harrison

**Affiliations:** 1 Centre for Health Systems and Safety Research, Australian Institute of Health Innovation, Macquarie University, Level 6, 75 Talavera Road, North Ryde, NSW Australia; 2 Behavioural Sciences Unit, School of Clinical Medicine, Discipline of Paediatrics & Child Health, UNSW Medicine and Health, Randwick Clinical Campus, UNSW Sydney, Sydney, NSW Australia; 3 Kids Cancer Centre, Sydney Children’s Hospital, Sydney, NSW Australia; 4 Sydney Youth Cancer Service, Nelune Comprehensive Cancer Centre, Prince of Wales Hospital, Sydney, NSW Australia; 5 School of Nursing and Midwifery, Monash University, Melbourne, Victoria Australia; 6 Sacred Heart Supportive and Palliative Care, St Vincent’s Hospital Sydney, Sydney, NSW Australia; 7 Australian Commission on Safety and Quality in Health Care, Sydney, NSW Australia; 8 Nafs Counselling, Sydney, NSW Australia; 9 Sisters’ Cancer Support Group Inc., Sydney, NSW Australia; 10 Centre for Health Systems and Safety Research, Australian Institute of Health Innovation, Macquarie University, North Ryde, NSW Australia; 11 District Clinical Governance, South Western Sydney Local Health District, Sydney, NSW Australia; 12 Macarthur Cancer Therapy Centre, South Western Sydney Local Health District, Sydney, NSW Australia; 13 Murray Primary Health Network, Bendigo, Victoria Australia; 14 Liverpool Hospital, South Western Sydney Local Health District, Sydney, NSW Australia

**Keywords:** Advance care planning, End-of-life care, Culturally and linguistically diverse, Person-centred care, Co-design

## Abstract

**Background:**

Advance care planning (ACP) describes the process of supporting individuals at any age or stage of health to consider and share their personal values, life goals, and preferences regarding future health care. Engaging in ACP is associated with better-quality of care in which people receive care in lines with their wishes, values and preferences. Direct translations of ACP guides and resources do not attend to the considerable inter- and intra-ethnic variations in cultural and religious or spiritual beliefs that shape preferences among people from culturally and linguistically diverse (CALD) backgrounds. ICanCarePlan is a three-year project that aims to determine the prevalence of ACP documentation among people from CALD backgrounds with cancer, identify resources available and their use to support ACP among CALD communities, identify barriers and facilitators of person-centred ACP, and to develop, through co-design with consumers and clinicians, approaches that enhance the process ACP for people from CALD backgrounds.

**Method:**

A mixed-method sequential approach will be used comprising of four studies. Study one is retrospective medical record review of approximately 1500 medical records to establish the prevalence of ACP documentation among CALD patient records in cancer services. Study two is a document analysis synthesising the resources available in the Australian health system to support ACP. Study three is a qualitative study with healthcare staff and consumers to explore barriers and enablers of person-centred ACP. Evidence generated from studies one to three will inform the conduct of co-design with stakeholders to develop approaches to improve ACP processes among CALD communities. Language, technical and financial support for meaningful involvement with consumers from CALD backgrounds throughout this project is outlined. A plan for distress management is also made due to sensitive nature of the topic. The research project has also established a project steering group consisting of three consumer members who are from CALD backgrounds.

**Discussion:**

The project will address a national priority issue for a growing population of CALD communities in Australia. The project will provide novel evidence of ACP among CALD communities and novel strategies developed with stakeholders to enhance uptake and experiences of ACP.

**Supplementary Information:**

The online version contains supplementary material available at 10.1186/s12904-024-01453-z.

## Introduction

Advance care planning (ACP) describes the process of supporting individuals at any age or stage of health to consider and share their personal values, life goals, and preferences regarding future health care [1–3]. ACP is a part of person-centred care; ACP should occur across the continuum of care, integrating specific values, goals and preferences with shared decision-making as care evolves [1, 2]. For people with life-limiting disease, ACP provides individuals with opportunities to extend their autonomy in decision making to stages of life in which they are unable to make decisions for themselves [4]. ACP aids families and health care providers to make informed choices and to develop care plans aligning with patients’ values, goals and personal preferences [5]. In enhancing person-centred care and decision-making, ACP is associated with reduced hospitalisation, intensive care unit admission, invasive medical procedures at the end of life, and reduced stress, anxiety and depression amongst patients and support persons [5–10]. As a result, ACP is ultimately associated with improved quality of life for patients and carers [4]. 

Culturally and linguistically diverse (CALD) is a term used in Australian context to describe people who are born overseas or have a parent born overseas, or who speak a language other than English at home or engage in a cultural or religious practices that are different to the mainstream practices [11, 12]. A narrative review of 43 studies published between 2017 and 2022 identified that culture, ethnicity and language proficiency influence opportunities for ACP, exposing CALD communities to potentially burdensome health care at the end of their life [13]. The review also identified that rate of uptake of ACP and the patient’s desire to talk and plan about death was related to length of stay in the destination country, acculturation and cultural and religious beliefs of ACP akin to defeat and competing with God [13]. Effective communication between healthcare staff and consumers (patients and their family or care support person) is an essential element of the ACP process [14, 15]. Limited proficiency in English language was identified as a key factor contributing to poor quality ACP; a systematic review of 10 studies examining the use of interpreters among people with cancer with low-English proficiency identified inadequate goals of care discussions when professional interpreters were not used [16]. 

ACP can take many forms; when formally documented, an Advance Care Directive (ACD) is a formal plan of a person’s wishes and preferences about their future care. ACP often occurs informally and includes any instance of information sharing between healthcare staff, patients and/or their support person about their wishes, values and preferences for care [5, 6, 17]. There is limited evidence of the uptake of any form of ACP among people from CALD backgrounds experiencing cancer; limited administrative socio-cultural data available in patient records is a key challenge for generating this evidence. One large prospective audit study of 4,187 records of adults aged 65 years and over showed that there was a lower prevalence of completed advance care directives among those born outside of Australia (21.9%) compared to those born in Australia (28.9%) [17]. Further, the extent to which socio-cultural variables such as language spoken at home, preferred language, interpreter use, country of birth or parent’s country of birth, religion and time spent in the country of residence collectively influence the uptake of ACP is unclear.

Intra- and inter-group differences in the preferences, customs, values and expectations of care towards the end of life are notable among diverse CALD populations, reinforcing the importance of ACP for CALD communities. Documented differences include sharing the burden of worry and/or caregiving, attitudes towards explicit talk about death and dying within clinical encounters, traditional approaches as part of supportive care, and desire to be cared for/to die at home [7, 8]. Yet healthcare staff and consumers have limited access to support them in engaging in ACP. Direct translations of ACP guides and resources do not attend to the range of cultural, religious or spiritual beliefs that shape preferences for end-of-life care (care delivered to the patient at the end of their life) for people from CALD backgrounds [18–20]. . Such guides may require cultural adaptation in their content and/or implementation – beyond direct language translation – to address the cultural and ethnic factors that shape preferences regarding end-of-life care and to support healthcare staff to engage in such communications. Without culturally and linguistically appropriate resources, people from CALD backgrounds may be left without the required support to engage in ACP and may be exposed to poor quality of care at the end of their life that is not aligned with their preferences, values or needs [21, 22]. 

The iCanCarePlan project aims to address this gap by supporting ACP among people with cancer from CALD backgrounds. The project aims to provide novel evidence of the uptake and experience of ACP among CALD communities, resources available to support staff and consumers, and to co-design evidence-based approaches to improve the process of ACP between healthcare staff and CALD populations .

### Objectives

1. To determine the uptake and nature of ACP amongst people from CALD backgrounds experiencing cancer and examine socio-cultural factors predicting the uptake.
2. To characterise the resources available to support ACP in the Australian health system context and determine the extent of their relevance for people from CALD backgrounds.
3. To explore barriers and facilitators to ACP amongst people from CALD backgrounds with cancer and their clinicians.
4. To co-design a new or adapted culturally appropriate strategy to support person-centred, high-quality ACP for people from CALD backgrounds experiencing cancer.

## Methods and analysis

### Study design

**Fig. 1:**
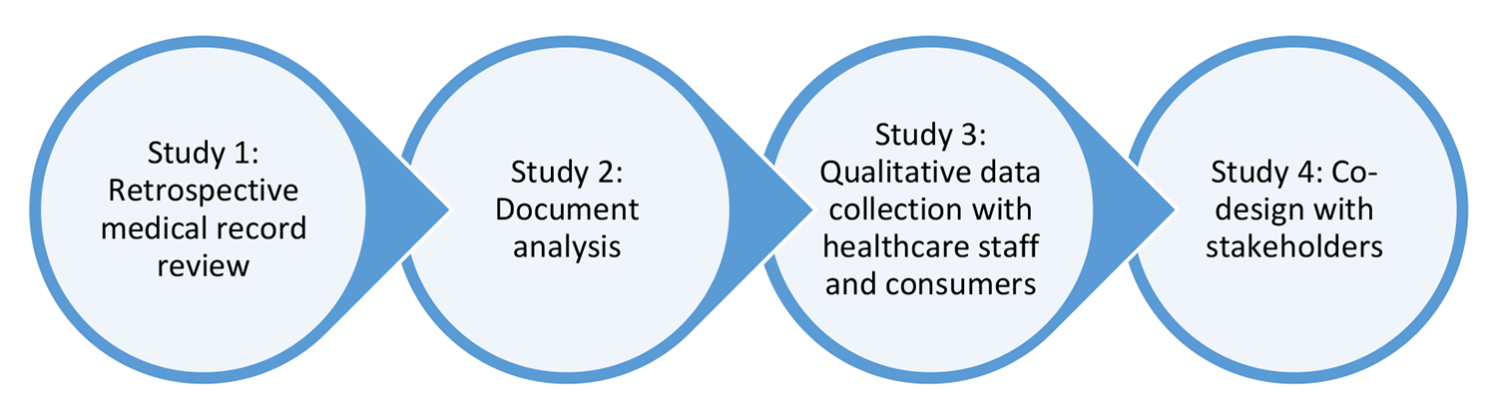
Pictorial representation of iCanCarePlan project comprising of four studies

A mixed method sequential approach will be used comprising four studies (Fig. 1). A mixed-method approach [23, 24] was selected to integrate information about the evidence of the extent of ACP (uptake and nature of ACP) among CALD populations, then identify resources available to support healthcare staff and consumers and the factors (barriers and facilitators) that have impact on ACP with CALD communities. This knowledge will inform a co-design process as the final study of the project. Co-design workshops will be conducted with consumer, policy and clinician stakeholders to collectively identify the target issue(s), design a solution(s) and develop a plan that will be used by the project team to implement the proposed solution(s).

## Study 1: retrospective medical record review

Retrospective medical record review will be used to address objective one as an established approach to data collection pertaining to ACP. This approach has been used in past studies to determine the prevalence of ACP documentation [5, 17]. This study methods will be guided by our prior medical record review research in Australian cancer services [25]. 

### Setting

Study 1 will be conducted across three health services providing comprehensive cancer and/or palliative care to patients from CALD backgrounds with cancer. These services provide care in inpatient, outpatient and in the community setting. The services are located in three distinct government administrative areas in the state of New South Wales that have large population of people from diverse CALD backgrounds [26]. These three health services have been purposefully selected as they attend to a diverse demographic of CALD communities [26]. 

### Sample

A total of 1500 medical records will be reviewed (500 at each site). This sample size is sufficient to address the study objectives. To determine the prevalence of ACP documentation based on estimated rate of 25% of ACP completion and 5% margin of error, a minimum sample size required is 300 records [27]. We will also explore association between prevalence of any ACP documentation with the seven socio-cultural variables that are recommended by the Australian Bureau of Statistics in their Standards for Statistics on Cultural and Language Diversity [28]. These seven variables are country of birth, language spoken at home, preferred language, interpreter required, year of arrival in Australia, religious affiliation and if one/both parents born overseas. Based on the formula *n* = 100 + 50(i) where (i) is the number of independent variables, minimal sample size required to examine the association is 450 medical records [29]. The sample size of 1500 is sufficient to explore the association between the presence of any ACP documentation and the seven socio-cultural variables outlined [29]. 

### Eligibility and recruitment

A medical record will be eligible for inclusion if: (a) it belonged to a person from CALD background (born outside of Australia or had any/both parents born outside of Australia, speaks a language other than English at home or requires an interpreter) AND (b) there is a diagnosis of cancer that has poor prognosis, AND (c) it belongs to a person with cancer who had their first episode of care between January 2017 – December 2020 (an ‘episode of care’ defined as being seen by a health professional at the participating health service for a cancer-related illness and/or management for the first time). No age restrictions will be in place. Inclusion of cancers with poor prognosis for adults (> 18 years of age) and children and young adults (< 18 years of age) is based on identification by Cancer Australia National Cancer Control Indicators (Supplementary File A) [30, 31]. Records that do not meet the eligibility criteria will be excluded.

### Data extraction

Each service will generate a list of patient records and the research team will apply the inclusion criteria to select eligible records using a random number generator. Once the eligible patient records are identified, a data extraction template (Supplementary File B) will be used to collect data. Each medical record will be retrospectively reviewed from the time of their first episode of care to the current date or until the date for when the data is available.

### Data analysis

Data analysis will be carried out using the R statistical software package. The primary outcome of interest will be the prevalence and type of ACP documentation (such as presence of an Advance Care Directive, resuscitation plan, conversation about goals of care or place of death) in the paper and/or electronic based medical records of patients with cancer from CALD backgrounds. Descriptive statistics (e.g., frequency, percent, sum, mean, median, standard deviation) will be used to describe the sample characteristics and frequency and nature of ACP documentation. Chi-square tests of independence will be used to compare key variables (such as age, sex, country of birth, language spoken at home, interpreter required, preferred language etc.) and logistic regression will be used to identify the association between the presence of ACP documentation to socio-cultural variables of the diverse CALD populations.

## Study 2: document analysis

Altheide’s document analysis approach will be used to address objective two, to characterise and explore the relevance of current resources available to healthcare staff and consumers from CALD backgrounds that support them during the process of ACP [15, 32, 33]. 

### Setting

The study will examine resources available at all levels of the Australian healthcare system. The websites of Australian government departments and health agencies at national, state and local health administrative levels will be searched. Relevant non-government organisations that have key focus on ACP, palliative and end-of-life care will be searched to identify and include eligible resources for analysis.

### Eligibility and inclusion

Eligible data sources will comprise of contemporary resources (available in print, audio or video format) published from January 2013 to June 2023. Resources that provide guidance for the communication process for ACP (for example: question guides, how to guides, fact sheets, toolkits, techniques for communication other similar documents) will be eligible for inclusion. Strategic plans, policy documents, frameworks and implementation plans will be excluded as they do not provide the information relevant to the study objective. Resources relating to education modules and professional development courses will also be excluded.

#### Data extraction

The data extraction will be completed in accordance with the iterative process of search, selection and extraction outlined in prior research using the Altheide’s approach [15]. Two researchers will complete an initial search of the eligible organisations (Supplementary File C) websites using key words (palliative care, end-of-life care, life support care, advance care directive, advance care planning, advanced personal plan, life wishes, substitute decision-maker, carer decision maker) to identify initial set of resources. These key words have been developed using collective knowledge and experience of the research team and relevant literature [34, 35]. These resources will then be subjected to inclusion criteria by the two same researchers who completed the initial search and eligible resources will be identified. Any discrepancies during this stage will be resolved through discussion with the project lead. Data extraction tool will be developed and used to collect consistent information from the eligible documents relevant to study objective. The two researchers will complete the data extraction using the data extraction tool for the subset of the resources. The findings will be compared and discussed in a meeting with the project lead. Following this, one researcher will complete the data extraction of the remaining eligible resources with findings discussed in regular meetings with the project lead.

### Data analysis

Narrative synthesis will be employed for evidence synthesis [15]. Contents of the eligible resources that provide guidance or support to consumers from CALD backgrounds or healthcare staff for the ACP will form the unit of analysis. The data extracted will be analysed to present the characteristics of the resources. Frequency, count and proportion of the resources will be presented under the categories of total number of resources, form (print, audio, video), source (government or non-government), target group (healthcare staff and/or consumers) and type of settings (such as primary, secondary, community, specialist or other). This will be followed by presenting a narrative description of operationalisation of ACP process with consumers from CALD backgrounds. We will examine the extent of cultural, religious or spiritual, language and other specific considerations for ACP with diverse CALD communities outlined or discussed in the included resources. We will also examine consumer involvement in development of the resources.

## Study 3: qualitative data collection with healthcare staff and consumers

Qualitative data in the form of focus groups or interviews with healthcare staff and consumers will be used to address the objective three. Focus groups and semi-structured interviews provide a flexible approach to data collection to obtain in- depth data on experiences and expectations of ACP [36, 37]. Given the nature of the discussion, some participants (especially consumers from CALD backgrounds) may not wish to participate in the focus groups. These participants will be provided the opportunity to participate via one-on-one semi-structured interviews with the researcher.

### Setting

Evidence of the current barriers and enablers to ACP and use of current resources with CALD communities experiencing cancer will be captured nationally through focus groups with healthcare staff and consumers from CALD backgrounds accessing cancer care.

### Sample

Number of the focus groups will be determined by emerging findings and point of saturation [36, 38]. Approximately four to six focus groups (two-three focus groups with healthcare staff and two-three with CALD consumers and consumer representatives), with a total of up to 48 participants will be conducted. Each focus group will consist of six to eight participants. This sample size is sufficient to allow in-depth discussion on the topic while managing the amount of information collected to analyse the data [36]. 

### Recruitment and eligibility

Healthcare staff working in cancer or palliative care for a minimum of six months, consumer representatives from consumer organisations that cater to cancer and palliative care, and consumers from CALD backgrounds who have used cancer or palliative care services will be eligible to participate in this study. All eligible participants will be 18 years of age or older and willing to provide consent for participation.

Participant recruitment and data collection will be an iterative process with focus groups and/or interviews conducted concurrently. Purposive sampling will be used to recruit participants through multiple channels (social media, networks and consumer organisations). Recruitment will be based on participant opt-in; contact details of the research team will be distributed through study advertisements (poster, social media posts) and potential participants will be provided with opportunity to contact research team first. Eligibility screen will be conducted, and eligible participants will be provided with study information sheet and consent form (PICF) prior to data collection. For participants who require language support during screening and recruitment, bilingual fieldworkers or interpreters will be used to convey the information and obtain consent as required. PICFs will be made available to participants in their preferred non-English language as needed.

Based on our previous work [39], we have established a project steering group that consists of three consumer members from CALD backgrounds with lived experience of cancer. Feedback from these three consumer members was sought for developing consumer facing research material such as the participation information sheet and recruitment advertisement to facilitate participation of consumers from CALD backgrounds in interviews or focus-groups and subsequent co-design workshops. These consumer members will also support the research team in developing a recruitment strategy for recruiting people from CALD backgrounds in this research.

### Data collection

Data collection will commence after the consent is obtained and recorded. An interview guide informed by thematic domains framework (TDF) will be used to guide the qualitative data collection [40, 41]. The TDF is selected to elicit responses from the participants to identify factors (barriers and facilitators) responsible for delivery of quality ACP with people from CALD backgrounds with cancer [42]. The focus groups/interviews will be conducted either online, face-to-face or using a hybrid model (combination of face-to-face and online participation) as suitable to the participants.

#### Data analysis

Data will be analysed using the Framework method [43]. TDF will be used as the framework for this analysis. Study data will be managed using NVivo [41]. The audio recording of the focus groups/interviews will be transcribed verbatim. Data will be transferred to NVivo, and analysis will be conducted in it. Data analysis will draw out common experiences and perceptions regarding the barriers and facilitators to ACP amongst people from CALD backgrounds experiencing cancer against the TDF framework. Data will be both inductively and deductively analysed. The data will be analysed and coded under the categories of TDF. A working analytical framework will be developed in the initial stage through an iterative process of re-reading and coding the transcripts between two researchers and the project lead. A Framework Matrix charting the final categories and codes will be developed. Following this, themes will be developed with preliminary themes refined in consultation with contribution from the wider research team.

## Study 4 – co-design approaches to improve quality ACP

**Fig. 2:**
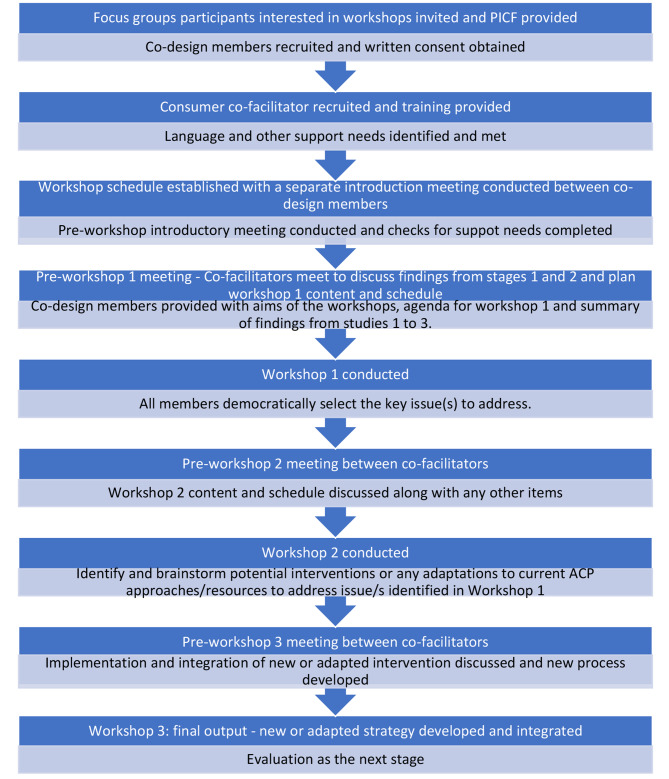
Co-design process

Adapted experienced-based co-design (EBCD) approach will be used to conduct this study. This approach has been used in our prior work to co-design health service interventions with relevant stakeholders including consumers from CALD backgrounds [39, 44]. Adapted EBCD consist of two adaptations made to the co-design approach. These two adaptations are (a) inclusion of a preparatory stage to identify and meet various support needs of the co-design members and (b) inclusion of a consumer co-facilitator to mitigate power imbalance during co-design workshops [39, 44]. These two adaptations aim to improve the meaningful participation of co-design members and improve their experiences of co-design process [39]. Following the preparatory phase, a series of three co-design workshops will be conducted accompanied by an introductory meeting between all co-design members and a set of preparatory meetings in between co-facilitators prior to each workshop (Fig. 2.).

### Preparatory work prior to co-design

Preparatory work will be undertaken to support meaningful participation of consumers from CALD backgrounds in co-design workshops [39, 45]. This involves identifying and meeting the support needs of the co-design members and ensuring equal power distribution between all members during co-design.

### Identifying support requirements

Based on our prior work [39, 44, 45], language, technological and financial support needs of the co-design members will be identified and addressed to enhance equal participation of all co-design members in the co-design workshops [45]. Professional interpreters or bilingual research fieldworkers will be used to provide language support as required by potential participants. Through our existing consumer co-facilitator network [46], we have access to bilingual research fieldworkers who can speak 11 languages. The members of this network are trained in conducting research and have knowledge of the research project. In a case where the language support is not provided with bilingual research fieldworker, an interpreter will be used. The mode of meeting (online, face to face or hybrid) will be determined based on the preferences of co-design members. Technical support will be provided via one-to-one communication with participants to facilitate online participation in the co-design workshops where necessary. Consumers and their representatives will be remunerated for their time to take part in the project appropriate to the nature of their roles based on the rates proposed by Health Consumers New South Wales [47, 48]. 

### Strategies to mitigate power imbalance during co-design

To enhance meaningful participation of consumers from CALD backgrounds and to minimise the power imbalance between researchers and consumers in co-design workshops, consumer co-facilitators will assist the research co-facilitator to conduct the co-design workshops [49]. Employing consumer and research co-facilitators in co-design encourages a more inclusive culture to encourage engagement and provide co-facilitators to adequately identify and address any issues and shifts in power dynamics [49]. Consumer co-facilitators will be provided a role description and receive training in the research topic and co-facilitation prior to the co-design workshops.

### Co-design procedure

#### Sample

Approximately 4–6 members (2–3 consumers/consumer representatives and two-three healthcare staff) will be involved as members of the co-design group [39, 50]. Based on our prior work with diverse consumers, this size of group membership provides an opportunity for all members to contribute fully and effectively including with use of interpreters and support persons [44]. The co-design group will also consist of research co-facilitators, a consumer co-facilitator and a bilingual fieldworker as needed relevant to the study population [39]. Those who have taken part in the focus groups/interviews will be requested to indicate their interest if they wish to be contacted about co-design workshops. Those who indicate willingness to be contacted will provide their email and telephone contact details for this purpose and be invited to take part as co-design members until 4–6 members have been recruited. The consumer co-facilitator will be recruited to co-facilitate each group via the existing consumer co-facilitator network. If any member withdraws from the co-design group, a new member will be invited to join the process. Recordings of the initial session(s) will be shared with the new members if they are joining late in the process. The change in co-design group members in between workshops would not impact the validity of the process but may introduce broader range of perspective enhancing the process. Written consent will be obtained prior to the conduct of co-design workshops.

### Co-design workshops

The codesign groups will be convened to adapt, design and implement solutions to the priority issues identified through studies one to three. Written informed consent will be obtained prior to the conduct of co-design workshops. A suitable place to conduct the workshops will be identified in consultation with the co-design members. The co-design group will meet for no more than 10 h in total; approximately for 2.5 h for three times over a period of six weeks. The duration and timing of the groups will be determined by the members to ensure suitability. A separate introductory meeting will be conducted between the group members for 30-minute prior to first co-design workshop. In this meeting, the group will develop terms of reference that will determine their ways of working and the preferred mode of meeting (online, face to face or hybrid) and meeting duration and frequency. Co-facilitators will meet prior to each co-design workshop to determine the scope and content of each workshop based on the progress to that point. The co-facilitators will also contact co-design members in between workshops to address any queries or concerns or to receive any feedback that members were not able to provide in the workshop. Preparatory meetings in between the workshops will allow co-facilitators to integrate theory into the content of the subsequent workshops [51]. 

## Discussion

### Ensuring study quality

The programme of work has been through independent peer review process as part of the Cancer Institute New South Wales Career Development Fellowship grant awarded to project lead (RH). This programme of work is funded based on the scientific quality of the proposal. The funding also requires an annual progress report to the funder. To further ensure the study quality, a project reference group has been established that meets twice in a year. The reference group consist of three consumer members with lived experience of cancer along with members from relevant policy, research, consumer interest and cancer and palliative care relevant organisations. Terms of reference have been co-developed with member of the project reference group. The main function of the reference group is to provide oversight on the project processes and progress against the research objectives.

### Ethics

The research project has received ethics approval from two National Health and Medical Research Council (NHMRC) accredited Human Research Ethics Committees (Reference number: 2023/ETH00216 and 520,231,235,452,528) by South-Western Sydney Local Health District Human Research Ethics Committee and Macquarie University Human Research Ethics Committee. Through the ethics applications and approvals, various ethical considerations have been explored and a risk mitigation plan created for each matter employing three main strategies.

Firstly, recognising the complexity of the programme of work involved, a research team consisting of project lead, project manager and associate investigators has been established. The research team will provide accountability for the conduct of the research activities. Secondly, recognising involvement of consumers from diverse language and cultural backgrounds and risk associated with potential for ineffective communication, budget has been allocated for translating consumer facing material and employing bilingual fieldworkers or interpreters in the research activities.

Thirdly, recognising the sensitivity of the topic area, the discussion about ACP may bring about distress to potential participants during qualitative data collection and in co-design workshops. This issue may arise in group discussions or during one-on-one interviews [52]. A process will be implemented whereby a list of supportive organisations will be provided to the participants prior, during and after each focus group/interview and co-design workshops. At the start of each focus group or interview and co-design workshop, research team will inform participant to let the research team know if they experience any distress. Participants will also be informed that they do not have to answer every question, that they can take a break from discussion at any time if they wish and if online, they can close their camera or video application if they need to [53]. During the discussion, if the researcher think that the participant is experiencing distress, the researcher will pause the discussion and ask if the participant requires a break. The researcher will also offer the list of support organisations if the participant wishes to speak with a relevant organisation. Participant will be asked if they require a referral for further support or if they require the researcher or the research team to contact their local general practitioner [53]. After each discussion, the researcher will follow-up with participants and co-design members to check on them. The project team also includes a practising clinical psychologist who could provide referrals for further support as needed.

### Anticipated operational difficulties

The main anticipated difficulty relating to this program of work is recruitment of consumers from CALD backgrounds in qualitative data collection. Discussion about experiences and expectations of ACP may bring distress among consumers from any backgrounds, but particularly for people from CALD backgrounds where cultural meaning of disease such as cancer and death and dying is different to that of the non-CALD population [54, 55]. Further varied cultural practices may dictate consumers readiness to talk about their experiences of ACP. Perception of a cancer as a ‘shameful’, stigmatised disease may further complicate the recruitment process [56]. To mitigate this issue, an iterative collaborative approach will be used. The researchers will identify and partner with community representatives, leaders and organisations working closely with various CALD communities to identify a recruitment strategy that would work for recruiting consumers from a particular community. This approach will be undertaken with help of the project steering group members, especially consumer members, who will assist with identification of these networks and introduce the research team where necessary. Through these connections, avenues for distributing recruitment material to recruit participants from CALD background and suitable approach for data collection (e.g. via focus group or on-one interview and most appropriate modality) will be determined. The iterative collaborative approach of working with consumer members of the project steering group and community representatives, leaders and organisations may also assist in identifying priority CALD communities to conduct language, culture or religion specific focus-groups or interviews. This approach will allow us to generate knowledge of the issues that may pertain to range of CALD populations but also be specific for some CALD communities.

### Potential research, policy and practice implications

The Australian National Consensus Statement on essential elements for safe and high quality end-of-life care highlights ACP as an essential element of person-centred high quality end-of-life care [57]. Provision of culturally appropriate resources and communication have been highlighted in the Consensus Statement for achieving safe and high quality end-of-life care for people from CALD backgrounds [57]. Need for meaningful ACP conversations with priority population groups such as those from CALD backgrounds have also been identified as a national information priority by Australian Institute of Health and Welfare considering increasing population of people from diverse CALD backgrounds [58]. 

This program of work will contribute to advancing research, policy and practice for meaningful ACP with CALD communities in the following four ways. The medical record review study will provide a real-world data on ‘what is happening’. This study will identify gaps for communities for ACP processes and documentation. The document analysis study will help understand the current state of ‘what is available’. This study will identify gaps in the resources and where the adaptation or new strategy are required to support healthcare staff or consumers to facilitate meaningful ACP. Qualitative data collected through focus groups or interviews will inform ‘how the conversations are happening’. This study will help understand needs, desires, barriers and facilitators for meaningful ACP and will directly inform how the strategy need to be tailored to suit the needs of CALD populations. Co-design will then help partner with end service users to directly shape the strategy to improve ACP that they receive. The co-designed strategy developed will be reported in the context of quality ACP issue addressed, the adaptation made to the process, the population who co-designed the strategy and the target population for whom the co-designed strategy is relevant for. The co-design strategy will also report on potential process and outcome success measures. Application of the strategy to address diverse cultural needs will be discussed along with the need for future adaptations to suit diverse CALD populations. The plan for implementation and evaluation of the strategy will follow as next steps separate to this programme of work.

## Conclusion

The project will address a national priority issue for a growing population of CALD communities in Australia. The project will provide novel evidence of ACP among CALD communities and novel strategies developed with stakeholders to enhance uptake and experiences of ACP. communities in Australia. The project will also provide important process related knowledge for enhancing participation of consumers from CALD communities in research concerning sensitive topics. Implementation and evaluation of the co-designed approach will be the next step.

### Electronic supplementary material

Below is the link to the electronic supplementary material.

Supplementary Material 1

## Data Availability

No datasets were generated or analysed during the current study.
