# Supplementary material for: Co-designing strategies to improve advance care planning among people from culturally and linguistically diverse backgrounds with cancer: iCanCarePlan study protocol
Source: BMC Palliat Care. 2024 May 18;23:123. doi: 10.1186/s12904-024-01453-z (PMC11102140; doi:10.1186/s12904-024-01453-z)
Supplement: Supplementary file 1 — Supplementary Material 1 [file 12904_2024_1453_MOESM1_ESM.docx]

**Supplementary file A -** Cancer Types with poor prognosis (based on 5-year survival rate)

| **Cancer types in adults (> 18 years of age)** | **Cancer types in children and young adolescents (<18 years of age)** |
| --- | --- |
| 1. Lung Cancer 2. Pancreatic Cancer 3. Oesophageal Cancer 4. Bladder Cancer 5. Brain Cancer 6. Liver cancer 7. Ovarian cancer 8. Cancer of unknown primary insitu | 1. Advanced Medulloblastoma and other CNS embryonal tumours and other Brain cancers 2. Metastatic neuroblastoma 3. Metastatic acute lymphoblastic leukaemia 4. Metastatic Acute Myeloid Leukemia 5. Metastatic Ewing sarcoma 6. Hepatoblastoma 7. Sarcomas (Rhabdomyosarcoma, Osteosarcoma & Non-rhabdomyosarcoma soft tissue sarcoma) |

**Supplementary File B -** Data extraction template

| **Variable/Indicator** | **Value** |
| --- | --- |
| Patient ID | xx |
| Health Service ID | xx |
| Sex | xx |
| Year of Birth | xxxx |
| Cancer Type | xx |
| Year of Diagnosis | xxxx |
| Date of first episode of care | xx/xx/xxxx |
| Date/Year of Death (if applicable/available) | xxxx |
| Country of Birth | xx |
| Preferred Language | English/Non-English/Not documented |
| Language spoken at home | English/Non-English/Not documented |
| Interpreter Required | Yes/No/Not documented |
| Religion | xx |
| Year of arrival in Australia | xxxx |
| Parents country of birth (if available) | xx |
| Date of documented conversation re EoL care preferences | xx/xx/xxxx |
| Documentation Type | Advance Care Directive/Advance Care Plan/Documented treatment preference for future care/Appointment of substitute decision maker/ED or hospital admission directive/Documented discussion of future care scenarios/Documented discussion of cultural, religious or spiritual preferences/Other |
| Who initiated the conversation/communication OR with whom this conversation occurred. | Patient/Care support person/Healthcare staff |
| Setting | Inpatient/Outpatient/ED/Community/Other |
| Total number of EoL care conversations/planning documentation | xx |
| Referral made to palliative care team | Yes/No |
| Date the referral made to palliative care team | xx/xx/xxxx |
| Was palliative care team involved | Yes/No |
| Date of first consultation with palliative care team | xx/xx/xxxx |
| Location of death | Home/Palliative care centre/Hospital ward/Other |
| Was location of death consistent with their preference | Yes/No/Not applicable |

**Supplementary File C -** List of organisations selected for document analysis.

| **Government Organisations** | **Non-government organisations and relevant programs** |
| --- | --- |
| Australian Commission on Safety and Quality in Health Care | Palliative Care Australia and associated state level organisations |
| Department of Health and Aged Care | CareSearch |
| New South Wales (NSW) Health | Paediatric Palliative Care |
| NSW Agency for Clinical Innovation | Royal Australian College of General Practitioners |
| Clinical Excellence Commission NSW | Multicultural communities councils |
| Cancer Institute NSW | Australia New Zealand Society of Palliative Medicine |
| Department of Health, Victoria | Palliative Care Nurses Australia |
| Safer Care Victoria | End of Life Directions for Aged Care (ELDAC) |
| Better Care Victoria | Red Kite |
| Department of Health Tasmania | Advance Care Planning Australia |
| Queensland Health | My Values Program |
| Australian Capital Territory Health Department | End of Life Essentials |
| Northern Territory Health Department |  |
| South Australia Health Department |  |
| Western Australia Health Department |  |
| Public Health Networks (PHNs) |  |
